# Triple metal-hypersensitivity in the patient with knee prosthesis: a rare case

**DOI:** 10.1186/2045-7022-5-S1-O2

**Published:** 2015-03-11

**Authors:** Galina Balakirski, Cecile Muehlhoff, Hans F Merk

**Affiliations:** 1 University Hospital of Aachen, Department of Dermatology and Allergology, Aachen, Germany

## Case presentation

A 70-years old healthy male patient presented in our clinic with a recurrent swelling, redness and limitation of movement of his right knee. Clinical examination and laboratory findings showed no evidence of bacterial infection of the joint. The patient had no other complaints.

According to the clinical history the patient received titanium prosthesis of the right knee in 2009. One year later he developed swelling, redness and limitation of movement of this joint. The patient reported also about a skin patch test that was performed and was positive for titanium. However there is no documentation available. In 2011 the prosthesis of the right knee was replaced by a CoCrMo/FeCrNiMnMoNbN/UHMWPE knee prosthesis due to persistent signs and symptoms. During the initial period of 6 months the patient reported to be free of complaints, but after this period he developed symptoms again. A CT-Scan of the right knee was unremarkable.

The new skin patch test was positive for cobalt(II)-chlorid (CoCl_2_) and potassium dichromate (K_2_Cr_2_O_7_). Five months later the skin patch test was repeated, but no reaction to CoCl_2_ or K_2_Cr_2_O_7_ was seen. The diagnosis remained questionable.

## Our clinical workup

Eight months after the last skin patch test the patient presented himself in our clinic. We repeated the skin patch test. We observed positive reaction to K_2_Cr_2_O_7_ after 72 and 168 hours. Reaction to other metals was negative. To strengthen the diagnostic value of our clinical workup we performed an ELISPOT with nickel(II)-sulfat (NiSO_4_), cobalt(II)-chlorid (CoCl_2_) and titanium(IV)-oxid (TiO_2_). The result was positive for all these substances. To reproduce the result we repeated the test 4 weeks later with the same outcome.

## Discussion

Metallic alloys are used in large number for osteosynthesis and long-term implants. Although considered as inert materials they have been shown to release both micrometric and nanometric particles (NPs) and debris in the surrounding body fluids and tissues which can cause health effects either at the implant site or in distant organs. Even the interaction between NPs and immune system has been demonstrated, the data available are limited. Nevertheless some NPs have been linked to allergic sensitization. There are already a few case reports about titanium-hypersensitivity, which could be proved in vitro. In our patient we could detect hypersensitivity for NiSO_4_, CoCl_2_ and TiO_2_ using ELISPOT.

## Consent

Written informed consent was obtained from the patient for publication of this abstract and any accompanying images. A copy of the written consent is available for review by the Editor of this journal.

